# Vitamin D Deficiency and Associated Factors Among Postpartum Women in Türkiye: A Primary Care Cross-Sectional Study

**DOI:** 10.3390/nu18203322

**Published:** 2026-10-10

**Authors:** Kamile Marakoğlu, Bayram Aldemir, Zeynep Ebru Şener, Bahadır Öztürk, Muslu Kazım Körez

**Affiliations:** 1 Department of Family Medicine, Faculty of Medicine, Selçuk University, 42130 Konya, Türkiye; ctflialdemir@gmail.com (B.A.); zesodan@gmail.com (Z.E.Ş.); 2 Department of Medical Biochemistry, Faculty of Medicine, Selçuk University, 42130 Konya, Türkiye; bahadirozturk@selcuk.edu.tr; 3 Department of Biostatistics, Faculty of Medicine, Selçuk University, 42130 Konya, Türkiye; mkkorez@gmail.com

**Keywords:** vitamin D, postpartum, breastfeeding, primary care, supplementation

## Abstract

**Background/Objectives**: The postpartum period involves substantial physiological changes that may affect maternal vitamin D status. This study aimed to determine serum 25-hydroxyvitamin D [25(OH)D] levels in postpartum women in Konya, Türkiye, and to identify associated factors. **Methods**: In this study, 25(OH)D levels were assessed in 267 women aged 18–49 who visited a family health center at 0–1, 6, 12, and 24 months postpartum. Sociodemographic, obstetric, dietary, and lifestyle data were collected using a structured questionnaire, and sun exposure, skin tone, and physical activity level were assessed using standardized methods. **Results**: Vitamin D deficiency (<20 ng/mL) was present in 86.9% of participants, and 56.2% reported regular vitamin D supplementation during lactation. In multivariable analysis, serum 25(OH)D levels were significantly higher at 0–1 months [exp(β) = 1.448, 95% CI: 1.165–1.799; *p* < 0.001] and 6 months postpartum [exp(β) = 1.340, 95% CI: 1.081–1.662; *p* = 0.008] compared with 12 months postpartum. Older age [exp(β) = 1.021, 95% CI: 1.004–1.037; *p* = 0.014], vitamin D supplementation during lactation [exp(β) = 1.190, 95% CI: 1.011–1.401; *p* = 0.036], summer enrollment [exp(β) = 1.441, 95% CI: 1.151–1.805; *p* = 0.001], and greater sun-exposed body surface area [exp(β) = 1.008, 95% CI: 1.001–1.016; *p* = 0.025] were independently associated with higher serum 25(OH)D levels. **Conclusions**: Vitamin D deficiency remains highly prevalent among postpartum women, with a substantial proportion not regularly using vitamin D supplements during lactation. These findings highlight the importance of promoting adherence to recommended supplementation and continued attention to maternal vitamin D status throughout the postpartum period.

## 1. Introduction

The postpartum period is a unique physiological stage characterized by substantial endocrine and metabolic adaptations that enable lactation while maintaining maternal calcium homeostasis [1,2]. During breastfeeding, approximately 200–300 mg of calcium is transferred daily into breast milk. To meet this increased calcium demand, coordinated endocrine adaptations occur, including increased secretion of parathyroid hormone-related protein (PTHrP), enhanced skeletal calcium resorption, suppression of parathyroid hormone secretion, and alterations in calcium–phosphate and vitamin D metabolism [2]. Although circulating 1,25-dihydroxyvitamin D levels decline after delivery from the elevated levels observed during pregnancy, adequate maternal vitamin D stores remain essential for maintaining maternal calcium homeostasis and ensuring sufficient vitamin D transfer to breast milk, both of which are critical for maternal and infant health [3,4].

The increased physiological demands of the postpartum period may increase women’s susceptibility to vitamin D deficiency [5,6]. Moreover, the burden of vitamin D deficiency differs substantially across geographic regions. According to the European Calcified Tissue Society position statement, vitamin D deficiency affects fewer than 20% of the population in Northern Europe, 30–60% of populations in Western, Southern, and Eastern Europe, and up to 80% of populations in Middle Eastern countries [7]. Likewise, a recent systematic review and meta-analysis confirmed a persistently high prevalence of vitamin D deficiency across Asia [8]. Despite abundant year-round sunlight in many low-latitude regions, vitamin D deficiency remains common because of limited sun exposure, cultural clothing practices, urbanization, obesity, and inadequate dietary intake [9,10].

In Türkiye, a meta-analysis including 40 studies and 111,582 individuals reported vitamin D deficiency prevalences of 63.0% in the general population, 63.5% among adults, 64.7% among women, and 76.0% among pregnant women [11]. Although several regional studies have described vitamin D status among postpartum women or mother–infant pairs in Türkiye, contemporary evidence remains scarce, and data on factors associated with maternal vitamin D status during the postpartum period are still limited [12]. Because postpartum women are routinely monitored in primary care settings, identifying women at increased risk of vitamin D deficiency may facilitate early recognition and targeted preventive interventions. Therefore, this study aimed to determine serum 25-hydroxyvitamin D [25(OH)D] levels among postpartum women attending a primary care center in Konya, Türkiye, and to identify the factors associated with vitamin D status.

## 2. Materials and Methods

### 2.1. Study Design and Setting

This cross-sectional analytical study was conducted between 1 November 2024 and 31 December 2025 at a primary care center in Konya, Türkiye. Data were collected from postpartum women attending routine maternal and child health follow-up visits.

### 2.2. Ethical Considerations

Ethical approval was obtained from the Selçuk University Faculty of Medicine Ethics Committee (Approval No: 2024/453, Date: 10 September 2024). Written informed consent was obtained from all participants, and the study was conducted in accordance with the Declaration of Helsinki.

### 2.3. Participants and Sampling

The study population consisted of women attending the primary care center for routine infant and child follow-up visits corresponding to postpartum periods of 0–1, 6, 12, and 24 months, in accordance with the national follow-up program of the Turkish Ministry of Health. The corresponding assessment windows were 0–29 days for the 0–1-month group, 180–210 days for the 6-month group, 365–394 days for the 12-month group, and 661–760 days for the 24-month group [13]. All 267 eligible women attending these scheduled follow-up visits during the study period were consecutively invited to participate, and all provided written informed consent and were included in the final analysis, corresponding to a participation rate of 100%.

Sample size was calculated assuming a vitamin D deficiency prevalence of 78% among postpartum women, based on a previous Turkish study [14], a 95% confidence level, and a 5% margin of error. The minimum required sample size was 264 participants; the study was completed with 267 participants.

Inclusion criteria were age between 18 and 49 years, being in the postpartum period (0–1, 6, 12, or 24 months after delivery), and providing written informed consent. Women with conditions or medications affecting calcium or vitamin D metabolism, history of preeclampsia, malignancy, pregnancy loss (≥12 weeks), or multiple pregnancies were excluded.

### 2.4. Data Collection

**Figure 1:**
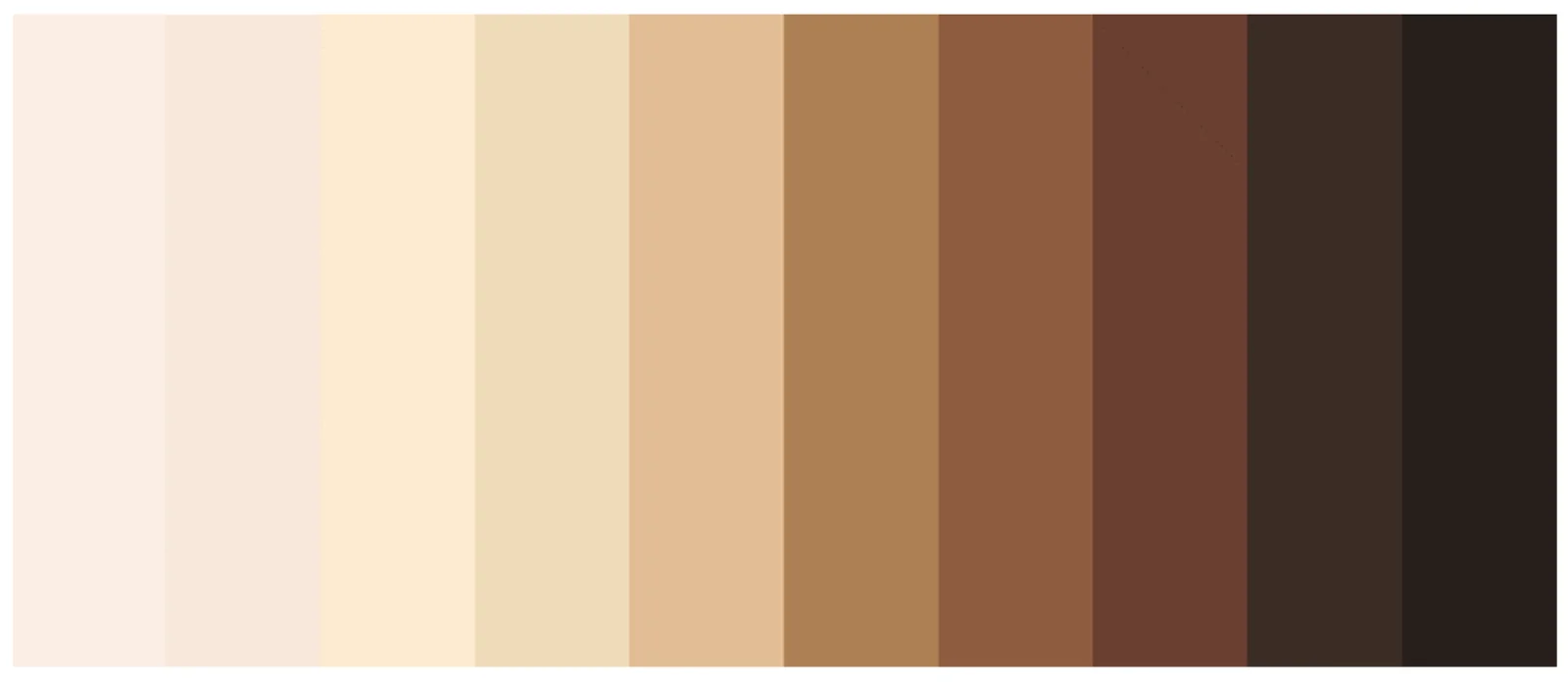
The Monk Skin Tone (MST) Scale used for skin tone assessment [16]. Reproduced from Monk E, “Monk Skin Tone Scale,” 2019, licensed under CC BY 4.0 (https://creativecommons.org/licenses/by/4.0/, (accessed on 6 October 2026)).

Data were collected through a structured face-to-face questionnaire administered by the researchers, covering sociodemographic characteristics (age, education, income, residence), reproductive history (parity, number of live births, breastfeeding status), and lifestyle factors (dietary habits, fish and dairy consumption, vitamin D and multivitamin supplementation, physical activity, sun exposure, sunscreen use, clothing style, and skin tone). Fish consumption was assessed according to weekly frequency (none, once, twice, or ≥3 times per week), whereas milk consumption was assessed according to daily intake (none, 1 glass, 2 glasses, or ≥3 glasses per day). Regular vitamin D and multivitamin supplementation and duration of use during pregnancy and lactation were recorded. For the regression analyses, breastfeeding status was categorized as currently breastfeeding versus not currently breastfeeding (stopped or never breastfed). Sun exposure was quantified using a sun exposure index calculated by multiplying the average weekly duration of sun exposure (hours/week) by the proportion of body surface area (BSA) exposed to sunlight, estimated according to participants’ clothing habits [15]. Skin tone was evaluated using the Monk Skin Tone (MST) Scale (Figure 1) [16]. Physical activity was assessed by recording the time spent over a 24-h period in five activity categories: resting, very light, light, moderate, and heavy activity. Basal metabolic rate (BMR) was estimated according to age and body weight using the Schofield equations, and total energy expenditure (TEE) was calculated by multiplying the time spent in each activity by its corresponding Physical Activity Ratio and hourly BMR. Physical Activity Level (PAL) was calculated as TEE/BMR and classified into three categories (sedentary/lightly active, active/moderately active, and vigorous/vigorously active) according to FAO/WHO/UNU recommendations [17].

### 2.5. Biochemical Analysis

Residual venous blood samples obtained during routine clinical evaluations were used. Samples were protected from UVB exposure, centrifuged, stored at −22 °C, and transported under cold chain conditions to the laboratory at 14-day intervals.

Serum 25-hydroxyvitamin D [25(OH)D] levels were measured using the Elecsys Vitamin D total III assay (Roche Diagnostics GmbH, Mannheim, Germany) on a Roche Cobas e 801 immunoassay analyzer based on the electrochemiluminescence immunoassay method. All analyses were performed at the Biochemistry Laboratory of Selçuk University Faculty of Medicine according to the manufacturer’s instructions, with routine internal quality control and instrument calibration procedures. Serum 25(OH)D levels were analyzed as a continuous variable. For descriptive purposes and to facilitate comparison with previous studies, vitamin D status was classified as deficiency (<20 ng/mL), insufficiency (20 to <30 ng/mL), and sufficiency (≥30 ng/mL), according to the Endocrine Society Clinical Practice Guideline [18].

### 2.6. Statistical Analysis

Statistical analyses were performed using R software (version 4.1.2; R Foundation for Statistical Computing, Vienna, Austria). Normality was assessed using the Shapiro–Wilk test and Q–Q plots, and homogeneity of variances was evaluated using Levene’s test. Continuous variables were presented as mean ± standard deviation (SD) or median (first quartile [Q1]–third quartile [Q3]), as appropriate, whereas categorical variables were presented as frequencies and percentages. Group comparisons were performed using independent-samples *t*-tests or one-way analysis of variance (ANOVA); Welch’s test was used when the assumption of homogeneity of variances was violated. Post hoc comparisons were performed using Tukey’s honestly significant difference (HSD) test following ANOVA and the Games–Howell test following Welch’s test. Correlations were assessed using Pearson’s or Spearman’s correlation coefficients, as appropriate. Serum 25(OH)D was analyzed as a continuous outcome. Factors associated with serum 25(OH)D levels were evaluated using univariable and multivariable generalized linear models (GLMs) with a gamma distribution and log-link function. Variables were selected for the multivariable model based on clinical relevance and/or a *p*-value < 0.20 in univariable analyses. To minimize multicollinearity, conceptually overlapping or highly correlated variables were not included simultaneously in the multivariable model. Breastfeeding duration was not included because of its strong correlation with postpartum period (r = 0.83). Multicollinearity was assessed using variance inflation factors (VIFs), with a maximum VIF of 3.60 in the final model. Results were reported as exponentiated regression coefficients (exp[β]) with 95% confidence intervals (CIs). A two-sided *p* < 0.05 was considered statistically significant.

## 3. Results

The 267 postpartum women had a mean age of 28.61 ± 5.46 years and a mean body mass index (BMI) of 27.47 ± 5.15 kg/m^2^. Vitamin D deficiency (<20 ng/mL) was found in 86.9% of the participants, insufficiency (20 to <30 ng/mL) in 10.1%, and sufficiency (≥30 ng/mL) in 3.0%. The mean serum vitamin D level was 11.98 ± 7.63 ng/mL. 25(OH)D levels differed across postpartum groups (*p* = 0.003), with the highest mean level observed at 0–1 month (14.16 ± 7.67 ng/mL) and the lowest at 12 months postpartum (9.83 ± 6.14 ng/mL). Serum 25(OH)D levels were significantly higher among women with university-level education and those who were employed (*p* = 0.039 and *p* < 0.001, respectively; Table 1).

Women using vitamin D supplements during lactation had higher mean 25(OH)D levels than non-users (13.51 ± 8.38 vs. 10.02 ± 6.02 ng/mL, *p* < 0.001). Women using multivitamin supplements during lactation had higher levels than non-users (13.46 ± 8.18 vs. 11.05 ± 7.13 ng/mL, *p* = 0.012). Mean 25(OH)D levels were significantly higher in summer than in spring (14.80 ± 8.51 vs. 10.63 ± 8.26 ng/mL, *p* = 0.028). No significant differences were observed for fish or milk consumption, vitamin D or multivitamin supplementation during pregnancy, sunscreen use, skin tone, or physical activity level (Table 2).

Breastfeeding duration showed the strongest inverse correlation with serum 25(OH)D levels (r = −0.229, *p* < 0.001), whereas greater sun-exposed body surface area was positively correlated with 25(OH)D levels (r = 0.200, *p* < 0.001). Positive correlations were also observed with the duration of vitamin D supplementation during lactation (r = 0.164, *p* = 0.007) and the duration of multivitamin supplementation during lactation (r = 0.157, *p* = 0.010) and pregnancy (r = 0.168, *p* = 0.006). In contrast, parity (r = −0.129, *p* = 0.036) and number of live births (r = −0.130, *p* = 0.034) showed weak inverse correlations with 25(OH)D levels. No significant correlations were observed with age, height, weight, BMI, age of the previous child, weekly sun exposure duration, or the sun exposure index (Table 3).

In univariable analyses, higher serum 25(OH)D levels were associated with university education, employment, the 0–1-month postpartum period, vitamin D and multivitamin supplementation during lactation, longer multivitamin supplementation during pregnancy, summer enrollment, and greater sun-exposed body surface area (BSA) (all *p* < 0.05). In contrast, longer breastfeeding duration, higher parity, and a greater number of live births were associated with lower serum 25(OH)D levels (all *p* < 0.05). Current breastfeeding status itself was not significantly associated with serum 25(OH)D levels in the univariable analysis (*p* = 0.705) (Table 4).

In the multivariable gamma regression model, age, postpartum period, vitamin D supplementation during lactation, season of enrollment, and sun-exposed BSA remained independently associated with serum 25(OH)D levels. Each additional year of age was associated with a 2.1% higher expected serum 25(OH)D level [exp(β) = 1.021, 95% CI: 1.004–1.037; *p* = 0.014]. Compared with women at 12 months postpartum, those at 0–1 month and 6 months postpartum had 44.8% [exp(β) = 1.448, 95% CI: 1.165–1.799; *p* < 0.001] and 34.0% [exp(β) = 1.340, 95% CI: 1.081–1.662; *p* = 0.008] higher expected serum 25(OH)D levels, respectively. No significant difference was observed at 24 months postpartum (*p* = 0.638).

Vitamin D supplementation during lactation was associated with a 19.0% higher expected serum 25(OH)D level [exp(β) = 1.190, 95% CI: 1.011–1.401; *p* = 0.036]. Compared with winter enrollment, summer enrollment was associated with a 44.1% higher expected serum 25(OH)D level [exp(β) = 1.441, 95% CI: 1.151–1.805; *p* = 0.001]. Each 1% increase in sun-exposed BSA was associated with a 0.8% higher expected serum 25(OH)D level [exp(β) = 1.008, 95% CI: 1.001–1.016; *p* = 0.025].

Current breastfeeding showed an inverse association after adjustment, although this did not reach statistical significance [exp(β) = 0.838, 95% CI: 0.697–1.007; *p* = 0.059]. Employment status (*p* = 0.075), parity (*p* = 0.077), education level, multivitamin supplementation during lactation, duration of multivitamin supplementation during pregnancy, and spring or autumn enrollment were also not independently associated with serum 25(OH)D levels (Table 4). Breastfeeding duration was not included in the multivariable model because of its strong correlation with postpartum period (r = 0.83), to minimize multicollinearity.

## 4. Discussion

This cross-sectional study, conducted among postpartum women attending a primary care center, revealed a high prevalence of vitamin D deficiency. The postpartum period is very a high-risk window for hypovitaminosis D because of increased physiological demands and lifestyle changes; however, primary-care studies comprehensively examining maternal vitamin D status and its determinants across this period remain scarce. The mean 25(OH)D level was 11.98 ng/mL, deficiency (<20 ng/mL) was present in 86.9% of participants, and only 3.0% had sufficient levels (≥30 ng/mL). This burden is consistent with reports from Türkiye and elsewhere identifying postpartum women as a particularly vulnerable group [12,14,19], although our prevalence was higher than that reported in some earlier studies, likely reflecting regional differences in lifestyle, clothing habits, limited sun exposure, and seasonal variation [10,20,21,22,23]. Although Konya is a sun-rich province, vitamin D deficiency remained highly prevalent, consistent with the regional ‘sunshine paradox,’ whereby clothing practices, predominantly indoor lifestyles, and limited supplementation may reduce the benefits of abundant sunlight [9]. Situated in central Anatolia, Konya provides a relevant regional setting for examining postpartum vitamin D status. These observations accord with the persistently high national prevalence of vitamin D deficiency, which the 2026 national expert consensus attributes, in part, to the limited implementation of mandatory food fortification [24]. The same consensus further notes that effective cutaneous synthesis in Türkiye is largely restricted to the interval between April and September [24]; accordingly, where sun exposure and supplementation remain inadequate, low 25(OH)D levels may persist well beyond the winter months—a pattern reflected in the deficiency observed across all four seasons in the present cohort.

Against this consistently low background, vitamin D status differed across postpartum periods. After adjustment for relevant covariates, women assessed at 0–1 and 6 months postpartum had significantly higher expected serum 25(OH)D levels than those assessed at 12 months, whereas levels at 24 months did not differ significantly from those at 12 months. In Türkiye, vitamin D supplementation of 1200 IU/day is recommended from the 12th gestational week through the end of the sixth postpartum month [25]. The higher 25(OH)D levels observed in our 0–1- and 6-month postpartum groups therefore coincide with the period covered by the national maternal supplementation program. However, the self-reported duration of vitamin D supplementation was generally shorter than the six-month postpartum period covered by the program, which may have limited its potential effectiveness in routine practice. Because individual supplement doses and reasons for discontinuation were not assessed, we could not determine whether the shorter duration reflected incomplete adherence or identify the factors underlying it. Moreover, vitamin D supplementation during lactation was independently associated with higher 25(OH)D levels in our study, consistent with evidence from a systematic review and meta-analysis of randomized clinical trials showing that maternal vitamin D supplementation during lactation improves maternal vitamin D status [19]. Together, these findings highlight the importance of continued attention to maternal vitamin D status throughout the postpartum period.

Reproductive and lactational factors may also contribute to differences in vitamin D status across the postpartum period. In univariable analyses, longer breastfeeding duration was associated with lower 25(OH)D levels, and higher parity showed a similar inverse association. Comparable findings have been reported, with lower vitamin D levels in breastfeeding women and multiparity identified as a factor associated with postpartum vitamin D deficiency [26,27], although some studies have found no such relationship [28]. These associations are biologically plausible, as lactation is accompanied by substantial adaptations in maternal calcium metabolism, including increased parathyroid hormone-related protein activity and mobilization of calcium from maternal bone to meet the demands of breast milk production [2,29]. With prolonged breastfeeding, these sustained physiological demands, particularly in the setting of inadequate vitamin D intake or supplementation, may contribute to maintaining low maternal vitamin D stores. Repeated pregnancies may similarly increase maternal nutritional demands when vitamin D stores are not adequately replenished between pregnancies. Overall, these findings suggest that reproductive history and the physiological demands of lactation may contribute to maternal vitamin D status during the postpartum period, although these effects are closely linked to postpartum timing and other maternal factors.

Whereas reproductive and lactational demands may contribute to lower maternal vitamin D status, supplementation represents an important modifiable factor. Vitamin D supplementation during lactation was associated with higher 25(OH)D levels, and this association remained significant after adjustment. This finding is consistent with trial and meta-analytic evidence showing that maternal vitamin D supplementation during lactation improves maternal vitamin D status [19,30]. Although multivitamin supplementation during lactation and longer duration of antenatal multivitamin supplementation were also associated with higher levels, these associations were not maintained after adjustment. Even so, 43.8% of our cohort reported no regular vitamin D supplementation during lactation, suggesting that primary care follow-up may provide an opportunity to reinforce recommended postpartum vitamin D supplementation and address potential barriers to continued use.

Environmental and behavioral factors were also relevant. Levels were higher in summer and among women with greater sun-exposed body surface area (BSA); notably, neither weekly sun-exposure duration nor the composite sun index was significant, whereas exposed BSA was—suggesting that how much skin is uncovered may reflect cutaneous synthesis more faithfully than recalled time outdoors, which is error-prone and may fall outside peak-UVB hours. Consistent with this, deficiency was more common among conservatively dressed women in Konya [23], and greater sun exposure predicted higher levels in Egyptian women [31]. Higher education and active employment were also associated with higher 25(OH)D levels in the initial analyses, consistent with previous studies reporting more favorable vitamin D status among employed and more highly educated women [32,33], although these associations were not maintained after adjustment. These socioeconomic associations may reflect differences in outdoor activity, lifestyle, and supplementation practices rather than direct biological effects [20,32]. Age was also independently associated with 25(OH)D levels, with a modest increase in expected levels with increasing age. This finding is consistent with a previous study among postpartum women in which younger maternal age (<30 years) was associated with vitamin D deficiency [27].

No significant associations were found between 25(OH)D and age of the previous child, sunscreen use, weekly sun exposure duration, fish or milk consumption, physical activity, skin tone, income, or residence. In contrast, summer enrollment, greater sun-exposed BSA, and vitamin D supplementation during lactation were associated with higher serum 25(OH)D levels in our study, consistent with previous reports highlighting the roles of season, sun exposure, and vitamin D supplementation in maternal vitamin D status [15,28].

This study has several strengths. Recruitment through primary care enabled the assessment of postpartum women during routinely scheduled maternal and child follow-up visits; recruitment of independent groups of women at four postpartum stages allowed variation across the postpartum period to be described; 25(OH)D was modeled as a continuous variable using gamma-distributed generalized linear models, avoiding information loss from categorization; and the assessment of sun exposure using both sun-exposed body surface area and a composite sun exposure index allowed environmental factors to be evaluated in greater detail.

Several limitations should be considered when interpreting these findings. First, the cross-sectional design precludes causal inference and does not allow assessment of within-woman changes in vitamin D status over time. Second, recruitment from a single primary care center may introduce some degree of selection and limit the generalizability of the findings to other postpartum populations. Third, detailed information on the specific vitamin D preparations, individual vitamin D doses, and the vitamin D content of multivitamin supplements was not available, limiting a more comprehensive evaluation of supplementation practices. No comprehensive dietary assessment or validated dietary questionnaire was administered; dietary data were limited to self-reported fish and milk consumption, while other potential dietary sources of vitamin D, such as eggs and fortified foods, were not assessed. These self-reported dietary and supplementation data may also be subject to recall bias. Finally, serum calcium and parathyroid hormone levels were not measured, precluding a more comprehensive assessment of calcium–vitamin D homeostasis.

## 5. Conclusions

Vitamin D deficiency remained highly prevalent among postpartum women. In adjusted analyses, serum 25(OH)D levels were significantly higher at 0–1 and 6 months postpartum than at 12 months, whereas levels at 24 months did not differ significantly from those at 12 months. Vitamin D supplementation during lactation, summer enrollment, and greater sun-exposed body surface area were also independently associated with higher serum 25(OH)D levels. These findings indicate that vitamin D deficiency remains an important concern throughout the postpartum period and highlight the importance of continued attention to maternal vitamin D status. Prospective and interventional studies are needed to clarify the factors contributing to postpartum vitamin D status and to determine the optimal duration and effectiveness of postpartum supplementation strategies.

## Figures and Tables

**Table 1:** Serum 25(OH)D Levels by Sociodemographic and Reproductive Characteristics of Postpartum Women.

Variable	*n* (%)	Serum 25(OH)D (ng/mL) Mean ± SD	*p*-Value
Education level			
Primary school or below	27 (10.1)	10.55 ± 7.67	**0.039** ^2^
Secondary school	74 (27.7)	10.82 ± 6.89 ^a^	
High school	88 (33.0)	11.25 ± 6.02	
University or above	78 (29.2)	14.40 ± 9.30 ^b^	
Occupation			
Housewife	233 (87.3)	11.35 ± 7.13 ^a^	**<0.001** ^1^
Civil servant	20 (7.5)	14.97 ± 7.17	
Other *	14 (5.2)	18.14 ± 12.07 ^b^	
Employment status			
Not working	232 (86.9)	11.38 ± 7.15	**<0.001** ^3^
Working	35 (13.1)	15.94 ± 9.42	
Income status			
Low	6 (2.3)	9.23 ± 4.61	0.456 ^1^
Middle	203 (76.0)	12.27 ± 7.86	
High	58 (21.7)	11.27 ± 6.98	
Residence			
Rural	41 (15.4)	12.06 ± 6.86	0.946 ^3^
Urban	226 (84.6)	11.97 ± 7.77	
Postpartum period			
0–1 month	99 (37.1)	14.16 ± 7.67 ^a^	**0.003** ^1^
6 months	63 (23.6)	11.08 ± 6.09	
12 months	52 (19.5)	9.83 ± 6.14 ^b^	
24 months	53 (19.8)	11.08 ± 9.48	
Breastfeeding status			
Never breastfed	3 (1.1)	26.43 ± 4.20 ^a^	**0.003** ^1^
Stopped breastfeeding	73 (27.3)	11.10 ± 8.76 ^b^	
Currently breastfeeding	191 (71.5)	12.09 ± 6.97 ^b^	
Total	267 (100.0)		

Values are presented as *n* (%) for categorical variables and mean ± SD for serum 25(OH) D levels. SD, standard deviation. * Including farmers, retirees, and workers. ^1^ One-way analysis of variance (Tukey HSD post hoc); ^2^ Welch’s test (Games–Howell post hoc); ^3^ Independent-samples *t*-test. Means with different superscript letters (a, b) within the same variable differ significantly (post hoc test, *p* < 0.05). Bold *p*-values denote statistical significance (*p* < 0.05).

**Table 2:** Serum 25(OH) D Levels by Lifestyle, Dietary, and Sun-Exposure Characteristics of Postpartum Women.

Variable	*n* (%)	Serum 25(OH)D (ng/mL) Mean ± SD	*p*-Value
Fish consumption (weekly)			
None	145 (54.3)	12.18 ± 7.40	0.651 ^3^
Once or more weekly	122 (45.7)	11.75 ± 7.91	
Milk consumption (daily)			
None	171 (64.0)	12.15 ± 8.16	0.628 ^3^
One or more glass	96 (36.0)	11.68 ± 6.60	
Vitamin D supplementation (lactation)			
No	117 (43.8)	10.02 ± 6.02	**<0.001** ^2^
Yes	150 (56.2)	13.51 ± 8.38	
Vitamin D supplementation (pregnancy)			
No	17 (6.4)	13.35 ± 7.87	0.445 ^3^
Yes	250 (93.6)	11.89 ± 7.62	
Multivitamin supplementation (lactation)			
No	164 (61.4)	11.05 ± 7.13	**0.012** ^3^
Yes	103 (38.6)	13.46 ± 8.18	
Multivitamin supplementation (pregnancy)			
No	21 (7.9)	10.21 ± 6.21	0.267 ^3^
Yes	246 (92.1)	12.13 ± 7.72	
Season of enrollment			
Winter	87 (32.6)	11.47 ± 6.82	**0.028** ^1^
Spring	81 (30.3)	10.63 ± 8.26 ^a^	
Summer	42 (15.7)	14.80 ± 8.51 ^b^	
Autumn	57 (21.3)	12.62 ± 6.71	
Sunscreen use			
No	127 (47.6)	11.47 ± 7.14	0.301 ^3^
Yes (SPF 30–50)	140 (52.4)	12.44 ± 8.04	
Monk Skin Tone Scale Score			
3	38 (14.2)	12.13 ± 6.77	0.086 ^1^
4	115 (43.1)	13.34 ± 8.72	
5	95 (35.6)	10.40 ± 6.46	
6	16 (6.0)	10.95 ± 4.98	
7	3 (1.1)	13.33 ± 13.10	
Physical activity level			
Sedentary/lightly active	124 (46.4)	12.31 ± 8.06	0.192 ^1^
Active/moderately active	95 (35.6)	12.14 ± 7.32	
Vigorous/vigorously active	48 (18.0)	10.81 ± 7.08	

Values are presented as *n* (%) for categorical variables and mean ± SD for serum 25(OH)D levels. SPF, sun protection factor; SD, standard deviation. ^1^ One-way analysis of variance (Tukey HSD post hoc); ^2^ Welch’s *t*-test; ^3^ Independent-samples *t*-test. Means with different superscript letters (a, b) within the same variable differ significantly (post hoc test, *p* < 0.05). Bold *p*-values denote statistical significance (*p* < 0.05).

**Table 3:** Correlations Between Continuous Variables and Serum 25(OH)D Levels.

Variable	Mean ± SD or Median (Q1–Q3)	Correlation Coefficient (r/ρ)	*p*-Value
Age (years)	28.61 ± 5.46	0.083	0.176 ^1^
Height (cm)	161.48 ± 5.89	0.002	0.971 ^1^
Weight (kg)	71.52 ± 16.17	−0.007	0.907 ^1^
BMI (kg/m^2^)	27.47 ± 5.15	−0.004	0.944 ^1^
Parity (per birth)	2 (1–3)	−0.129	**0.036** ^2^
Number of live births	2 (1–3)	−0.130	**0.034** ^2^
Age of previous child (months) ^a^	42 (23–78)	0.078	0.457 ^2^
Breastfeeding duration (months)	6 (1–12)	−0.229	**<0.001** ^2^
Vitamin D supplementation duration, lactation (months)	1 (0–2)	0.164	**0.007** ^2^
Vitamin D supplementation duration, pregnancy (months)	6 (4–6)	0.120	0.051 ^2^
Multivitamin supplementation duration, lactation (months)	0 (0–1)	0.157	**0.010** ^2^
Multivitamin supplementation duration, pregnancy (months)	6 (4–6)	0.168	**0.006** ^2^
Sun-exposed body surface area (BSA, %)	16 (9–20)	0.200	**<0.001** ^2^
Weekly sun exposure duration (hours)	14 (9–21)	−0.083	0.175 ^2^
Sun exposure index	1.96 (1.12–3.36)	0.109	0.077 ^2^

Values are mean ± SD for normally distributed variables and median (Q1–Q3) for non-normally distributed variables. Q1, first quartile; Q3, third quartile; SD, standard deviation. ^a^ Assessed only in multiparous women with a previous child (*n* = 93). ^1^ Pearson’s correlation coefficient; ^2^ Spearman’s rank correlation coefficient. Bold *p*-values denote statistical significance (*p* < 0.05).

**Table 4:** Univariable and Multivariable Generalized Linear Models of Factors Associated with Serum 25(OH)D Levels.

Variable	Univariable exp(β) (95% CI)	*p*-Value	Multivariable exp(β) (95% CI)	*p*-Value
Age, per year	1.010 (0.996–1.025)	0.146	1.021 (1.004–1.037)	**0.014**
Education level (ref: primary or below)				
Secondary	1.025 (0.780–1.348)	0.858	1.005 (0.776–1.301)	0.971
High school	1.066 (0.816–1.393)	0.637	1.077 (0.825–1.406)	0.586
University	1.364 (1.040–1.790)	**0.024**	1.148 (0.867–1.519)	0.336
Occupation (ref: housewife)				
Civil servant	1.319 (0.992–1.753)	0.056	–	–
Other	1.598 (1.142–2.236)	**0.006**	–	–
Employment (ref: not working)				
Working	1.400 (1.120–1.749)	**0.003**	1.233 (0.979–1.552)	0.075
Parity (per birth)	0.926 (0.868–0.988)	**0.019**	0.934 (0.866–1.007)	0.077
Number of live births	0.929 (0.870–0.992)	**0.027**	–	–
Postpartum period (ref: 12 months)				
0–1 month	1.441 (1.164–1.785)	**<0.001**	1.448 (1.165–1.799)	**<0.001**
6 months	1.128 (0.893–1.425)	0.309	1.340 (1.081–1.662)	**0.008**
24 months	1.128 (0.884–1.439)	0.329	1.056 (0.841–1.327)	0.638
Breastfeeding (ref: not currently breastfeeding)				
Currently breastfeeding	1.033 (0.872–1.225)	0.705	0.838 (0.697–1.007)	0.059
Breastfeeding duration (per month)	0.986 (0.975–0.997)	**0.011**	–	–
Vitamin D supplementation (lactation)	1.348 (1.162–1.565)	**<0.001**	1.190 (1.011–1.401)	**0.036**
Duration of vitamin D supplementation (lactation), per month	1.029 (0.992–1.067)	0.120	–	–
Multivitamin supplementation (lactation)	1.218 (1.042–1.424)	**0.013**	1.095 (0.926–1.295)	0.290
Duration of multivitamin supplementation (lactation), per month	1.042 (0.995–1.091)	0.078	–	–
Duration of multivitamin supplementation (pregnancy), per month	1.053 (1.016–1.091)	**0.005**	1.024 (0.990–1.060)	0.167
Season of enrollment (ref: winter)				
Spring	0.927 (0.763–1.126)	0.442	1.146 (0.952–1.381)	0.151
Summer	1.290 (1.018–1.636)	**0.034**	1.441 (1.151–1.805)	**0.001**
Autumn	1.100 (0.887–1.364)	0.382	1.190 (0.967–1.463)	0.100
Sun-exposed body surface area (BSA), per 1%	1.010 (1.002–1.018)	**0.016**	1.008 (1.001–1.016)	**0.025**

Values are presented as exponentiated regression coefficients [exp(β)] with 95% confidence intervals (CIs) from generalized linear models with a gamma distribution and log-link function (*n* = 267). Variables were selected for the multivariable model based on clinical relevance and univariable associations. To minimize multicollinearity, conceptually overlapping or highly correlated variables were not simultaneously included in the multivariable model. Employment status was selected instead of occupation, parity instead of number of live births, and supplementation status instead of the corresponding duration variables. Breastfeeding duration was not included because of its strong correlation with the postpartum period (r = 0.83). – not included in the multivariable model; BSA, body surface area; CI, confidence interval; ref, reference category. Bold values indicate *p* < 0.05.

## Data Availability

The data presented in this study are available on reasonable request from the corresponding author. The data are not publicly available due to ethical and privacy restrictions related to participant confidentiality.

## Abbreviations

The following abbreviations are used in this manuscript:

25(OH)D	25-hydroxyvitamin D
BSA	Body surface area
BMI	Body mass index
GLM	Generalized linear model
MST	Monk Skin Tone
Cl	Confidence interval
SD	Standard deviation
ANOVA	Analysis of variance
